# Supplementary material for: SMART-Q: An Integrative Pipeline Quantifying Cell Type-Specific RNA Transcription
Source: PLoS One. 2020 Apr 29;15(4):e0228760. doi: 10.1371/journal.pone.0228760 (PMC7190163; doi:10.1371/journal.pone.0228760)
Supplement: Attachment — Submitted filename: PONE-D-20-01686-comment.docx [file pone.0228760.s001.docx]

In this manuscript, the author developed an efficient and customizable analysis method, which can analyze gene transcripts in a cell type-specific manner. The method is facile and bring great convenience for users. I recommend its publication after some revisions as below:

1. The method is based on Z-stack images, RNA amplicons may merged together, or when target RNA has high expression, many amplicons may be too close to each other to be distinguished. The accuracy for RNA detection may be limited.
2. The author stated that their method has increased accuracy and quality control for the segmentation of nuclei and cells. However, they just showed the data for the segmentation of nuclei, while didn’t show the evidence of the segmentation of cells. Clinging cells always exist, and cell boundaries is more difficult to determined.
3. Another issue is how about the processing time SMART-Q required.
